# Regulation of PCSK9 Expression and Function: Mechanisms and Therapeutic Implications

**DOI:** 10.3389/fcvm.2021.764038

**Published:** 2021-10-15

**Authors:** Xiao-dan Xia, Zhong-sheng Peng, Hong-mei Gu, Maggie Wang, Gui-qing Wang, Da-wei Zhang

**Affiliations:** ^1^Department of Orthopedics, The Sixth Affiliated Hospital of Guangzhou Medical University, Qingyuan People's Hospital, Qingyuan, China; ^2^School of Economics, Management and Law, University of South China, Hengyang, China; ^3^Group on the Molecular and Cell Biology of Lipids, Department of Pediatrics, Faculty of Medicine and Dentistry, University of Alberta, Edmonton, AB, Canada

**Keywords:** lipid metabolism, cardiovascular disease, atherosclerosis, cancer immunotherapy, PCSK9, LDL receptor, major histocompatibility protein class I

## Abstract

Proprotein convertase subtilisin/kexin type 9 (PCSK9) promotes degradation of low-density lipoprotein receptor (LDLR) and plays a central role in regulating plasma levels of LDL cholesterol levels, lipoprotein(a) and triglyceride-rich lipoproteins, increasing the risk of cardiovascular disease. Additionally, PCSK9 promotes degradation of major histocompatibility protein class I and reduces intratumoral infiltration of cytotoxic T cells. Inhibition of PCSK9 increases expression of LDLR, thereby reducing plasma levels of lipoproteins and the risk of cardiovascular disease. PCSK9 inhibition also increases cell surface levels of major histocompatibility protein class I in cancer cells and suppresses tumor growth. Therefore, PCSK9 plays a vital role in the pathogenesis of cardiovascular disease and cancer, the top two causes of morbidity and mortality worldwide. Monoclonal anti-PCSK9 antibody-based therapy is currently the only available treatment that can effectively reduce plasma LDL-C levels and suppress tumor growth. However, high expenses limit their widespread use. PCSK9 promotes lysosomal degradation of its substrates, but the detailed molecular mechanism by which PCSK9 promotes degradation of its substrates is not completely understood, impeding the development of more cost-effective alternative strategies to inhibit PCSK9. Here, we review our current understanding of PCSK9 and focus on the regulation of its expression and functions.

## Introduction

Plasma low-density lipoprotein cholesterol (LDL-C) levels are positively correlated to the risk of cardiovascular disease (CVD). Statins, the currently most-prescribed lipid-lowering drug, reduce cardiovascular events by 20–40%. However, there is mounting evidence that about 50% of statin-treated patients and 80% of very high-risk patients do not achieve the recommended cholesterol values even with the highest tolerated dose. Furthermore, up to 20% of statin-treated people show statin intolerance, and about 10–12% of cases exhibit maladaptive side effects (1). Thus, there is an urgent need to develop a non-statin-based cholesterol-lowering drug.

**Figure 1:**
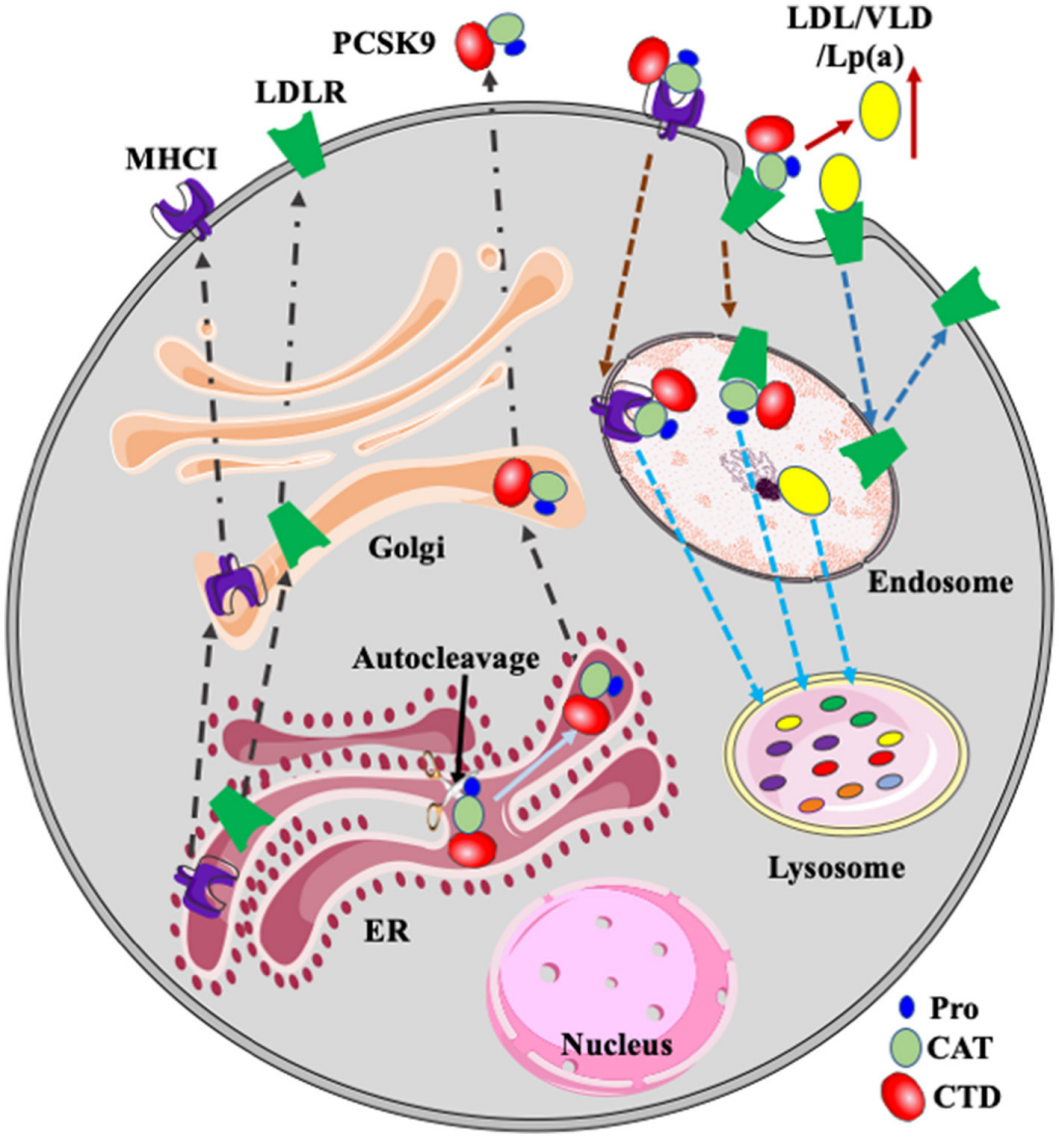
PCSK9, LDLR, and MHCI. PCSK9 is auto-cleaved in the ER. Mature PCSK9 is transported to the Golgi and then secreted. PCSK9 binds to LDLR and MHCI on the cell surface. After, the complex is delivered to endosomes *via* endocytosis and then transported to the lysosome for degradation. LDLR binds to its ligands such as LDL, VLDL, and Lp(a) and then the receptor/ligand complex enters cells *via* receptor-mediated endocytosis and is delivered to the endosome. In the acidic endosomal environment, the ligand, such as LDL, is released from LDLR and transported to the lysosome for degradation. LDLR is recycled to plasma membrane. PCSK9-promoted degradation of LDLR increases plasma levels of LDL and Lp(a). Pro, prodomain; CAT, catalytic domain; CTD, C-terminal domain.

Proprotein convertase subtilisin/kexin type 9 (PCSK9) plays a critical role in regulating plasma cholesterol homeostasis through promoting LDL receptor (LDLR) degradation (Figure 1). Gain-of-function mutations in PCSK9 cause autosomal dominant hypercholesterolemia, while loss-of-function mutations are associated with reduced plasma levels of LDL-C (2–6). PCSK9 also promotes major histocompatibility protein class I (MHCI) degradation and suppresses immune attacks to tumors (7) (Figure 1). Therefore, PCSK9 plays a central role in the pathogenesis of CVD and cancers. In addition, it has been reported that PCSK9, especially extra-hepatic PCSK9, can recruit inflammatory cells and induce local inflammation (8, 9). Here, we summarize the latest advances in PCSK9 and focus on its role in lipid metabolism and cancer immunotherapy and the molecular mechanisms for the regulation of PCSK9 expression.

## PCSK9 Function

Human and mouse PCSK9 is encoded by the *PCSK9/Pcsk9* gene located at chromosome 1p32.3 and 4C7, containing thirteen and twelve exons that encode a 692 and 694-amino acid PCSK9 protein, respectively (10). PCSK9 is highly conserved among mammals, including chimpanzee, monkey, camel, alpaca, rat, and mouse, with an approximate amino acid identity of 99, 96, 82, 81, 77, and 77%, respectively. The majority of identified gain-of-function and loss-of-function mutations occur in entirely conserved residues, such as gain-of-function mutations D35Y, L108R, S127R, D129G, N157K, R215H, F216L, R218S, A220T, R357H, D374Y, N425S, R468W, R496W and R499H, and loss-of-function mutations R104C, R105Q, G106R, G236S, L253F, G316C, N354I and S462P (2, 4, 11, 12). However, loss-of-function mutations R46L and R93C and gain-of-function mutations R96L, A168E, R499H, and S636R are not conserved between human and mouse or rat PCSK9. The correspondence residues of Arg46, Arg93, Arg96, Ala168, Arg499, and Ser636 in mouse/ rat PCSK9 are Pro49/Pro48, Gln93/Gln92, His99/His95, Thr211/Thr167, Trp512/Arg498, and Ser639/Ser635, respectively. Whether the difference in these residues affects PCSK9 function, however, is unclear.

PCSK9 contains a signal peptide [amino acid (aa) 1-30], a prodomain (Pro) (aa 31-152), a catalytic domain (CAT) (aa 153-425) and a Cys and His-rich C-terminal domain (CTD) (aa 426-692) (13) (Figure 1). The CAT contains a classical serine protease catalytic triad of Asp186, His226 and Ser386 and is highly conserved with the CAT of other proprotein convertases. PCSK9 is self-cleaved by the CAT at the FAQ152/SIPK site in the endoplasmic reticulum (ER). After autocleavage, the prodomain is associate with the CAT and masks the catalytic activity of PCSK9. This process is required for PCSK9 maturation and secretion. Compared to the other subtilisin-like serine protease family members, the CTD of PCSK9 is unique and contains multiple potential protein-protein interaction motifs (13). The CTD is positively charged and may interact with the negatively charged ligand-binding repeats of LDLR in the acidic endosomal environment, blocking recycling of the receptor (14, 15). In addition, partial deletion of the CTD markedly damages PCSK9 secretion, indicating its essential role in this process (16, 17). However, the underlying mechanism is unclear. Our recent study suggests that the CTD mediates PCSK9 secretion, possibly *via* a coat protein complex II (COPII) component Sec24 (17).

PCSK9 induces degradation of LDLR and its family members, including very low-density lipoprotein receptor (VLDLR), apolipoprotein E receptor 2 (ApoER2), and LDLR-related protein 1 (LRP1) (10, 18–20), thereby playing an essential role in lipid metabolism. However, PCSK9 at a physiological concentration can effectively degrade LDLR but not other LDLR family members in cultured cells (19, 20). Similarly, PCSK9 degrades LDLR but not LRP1 in mouse liver (21), but it can regulate visceral adipogenesis likely through promoting VLDLR degradation in mouse adipose tissues (22). Conversely, loss of functional PCSK9 in humans does not cause any known abnormality except for reduced plasma cholesterol levels (3, 6). Nevertheless, these findings indicate that PCSK9's action on its substrates is cell/tissue-type and/or species-dependent.

The CAT of PCSK9 directly binds to the epidermal growth factor precursor homology domain A (EGF-A) of LDLR on the cell surface. After endocytosis, PCSK9 remains bound to LDLR in the acidic endosome, preventing LDLR from recycling and redirecting the receptor to the lysosome for degradation (Figure 1) (19, 21, 23, 24). PCSK9 can also promote LDLR degradation *via* an intracellular pathway, especially when overexpressed in cultured cells (25). Of note, evolocumab and alirocumab are humanized monoclonal anti-PCSK9 antibodies targeting the catalytic domain of PCSK9. They block PCSK9 binding to LDLR on the cell surface and do not affect the intracellular pathway. On the other hand, Inclisiran is a small siRNA that targets PCSK9 mRNA and reduces its expression. It can inhibit both the intracellular and extracellular pathways. However, evolocumab, alirocumab, and Inclisiran all markedly reduce plasma LDL cholesterol levels in patients to a similar degree (26–28). Therefore, the extracellular pathway appears to be mainly responsible for the LDL-lowering effect of PCSK9 inhibition.

PCSK9 directs its substrates for lysosomal degradation (Figure 1), which does not require its proteolytic activity (29). Both caveolae-dependent and clathrin-mediated endocytosis have been reported to play an important role in the endocytosis of PCSK9/LDLR complex in HepG2 cells (30–32). Additionally, DeVay et al. reported that amyloid beta precursor-like protein 2 (APLP2) directly bound to PCSK9 and targeted the PCSK9/LDLR complex to lysosomes for degradation in HepG2 cells (33), while other studies showed that APLP2 did not affect PCSK9-promoted LDLR degradation in mice, HepG2, or Huh7 cells (34, 35). Differences in approaches and/or models used in these studies may cause these discrepancies. However, further studies are required to elucidate the underlying mechanism for PCSK9-promoted LDLR degradation.

### PCSK9 and LDL

Plasma LDL is eliminated from circulation primarily *via* hepatic LDLR. Upon binding, the LDL-LDLR complex is internalized *via* the clathrin-coated pits and subsequently delivered to endosomes, where LDL is released from LDLR and then transported to lysosomes for degradation, while LDLR is recycled to the cell surface to clear more LDL. Mutations in LDLR cause familial hypercholesterolemia (FH), characterized by elevated plasma levels of cholesterol, particularly LDL cholesterol, and increased risk of CVD (36). PCSK9 promotes LDLR lysosomal degradation. Circulating PCSK9 preferentially degrades LDLR in mouse liver (37), which may be due to hepatic heparan sulfate proteoglycans (HSPG). HSPG can recruit circulating PCSK9 to hepatocytes, enhancing its action on LDLR (38). Knockout of PCSK9 increases hepatic LDLR levels, reduces plasma LDL cholesterol levels, and improves sensitivity to statin treatment in mice (37).

LDL is derived from VLDL catabolism, which is a triglyceride-enriched lipoprotein exclusively secreted by the liver. Triglycerides in VLDL are hydrolyzed by lipoprotein lipase, resulting in intermediate-density lipoprotein (IDL), which can be further metabolized to LDL (39). PCSK9 can directly interact with apoB100, the main structural lipoprotein on VLDL, and inhibit apoB100 degradation, thereby promoting its secretion. Knockout of PCSK9 reduces hepatic apoB secretion and plasma LDL cholesterol levels in *Ldlr*^−/−^/*Apobec1*^−/−^ mice (40). Conversely, gain-of-function mutant PCSK9 increases apoB100 secretion in a rat hepatoma-derived cell line, McArdle-7777 cells (41). These findings indicate that, in addition to reducing the availability of hepatic LDLR, PCSK9 may promote the production of LDL through increasing secretion of VLDL. However, hepatocytes typically produce apoB100 in abundance, and the rate-limiting step in VLDL secretion is lipidation of apoB100. Therefore, the physiological role of PCSK9-promoted VLDL secretion may not be significant *in vivo*.

PCSK9 is also expressed in extra-hepatic cells and tissues, such as the vascular smooth muscles cells (VSMCs), macrophages, endothelial cells, the pancreas, the kidneys, the intestine and the central nervous system (10). The arterial vessel has the maximal secretion of PCSK9 at the lowest level of shear stress that occurs in the aortic branching and aorta-iliac bifurcation regions of the mouse aorta. Cultured VSMCs produce substantially more PCSK9 than endothelial cells (42). Elevated PCSK9 in VSMCs can reduce LDLR levels in VSMCs and macrophages (42, 43), which may impair LDL clearance and accelerate retention of LDL in VSMCs and macrophages in the location of arterial bifurcation. PCSK9 is also expressed in and secreted from pancreatic beta cells. However, inhibition of PCSK9 does not affect insulin secretion in the human EndoC-betaH1 beta cell line and mice even though PCSK9 promotes LDLR degradation in beta cells (44). Together, these findings indicate a cell-type-specific function of PCSK9.

### PCSK9 and Triglyceride-Rich Lipoproteins

Elevated plasma levels of triglyceride-rich lipoproteins and their remnants are an independent risk factor for atherosclerosis and CVD. Hepatic LDLR binds to apoE on remnant lipoprotein particles to mediate their clearance (36). Therefore, PCSK9 can affect plasma triglyceride and remnant cholesterol levels through the LDLR pathway. Elevated plasma PCSK9 levels are positively associated with plasma TG levels in humans upon a short-term high-fructose intake (45). Treatments with PCSK9 inhibitors increase clearance of VLDL remnants in patients (46). Alirocumab, a fully human PCSK9 monoclonal antibody, reduces LDL particles by 56.3% in human patients. This reduction is partly due to an increase in the clearance rate of IDL particles, thereby decreasing the conversion of IDL to LDL (27).

PCSK9 is expressed in the intestine and can affect chylomicron metabolism. Knockout of PCSK9 in mice significantly reduces lymphatic apoB48 secretion and increases secretion of TG-rich large chylomicrons. Clearance of chylomicron remnants is also increased in *Pcsk9*^−/−^ mice (47). Rashid et al. further demonstrated that PCSK9 promoted chylomicron secretion through both LDLR-dependent and -independent pathways in mice and a human enterocyte cell line, CaCo-2 cells, such as increasing the expression of apoB, microsomal triglyceride transfer protein and lipogenic genes in enterocytes (48). However, inhibition of PCSK9 by evolocumab or alirocumab does not significantly affect VLDL production or postprandial plasma levels of apoB48 and triglycerides in healthy humans or patients with hypercholesterolemia (49). Conversely, in patients with type-II diabetes mellitus, evolocumab reduces postprandial apoB48 levels even though the effect on postprandial triglyceride levels is not significant, while alirocumab can significantly reduce fasting plasma apoB48 and TG levels and postprandial TG levels (50). Plasma PCSK9 levels are also correlated with plasma apoB48-containing TG-rich lipoproteins in men with insulin resistance (51). However, the impact of PCSK9 on plasma levels of TG-rich lipoproteins, such as VLDL and chylomicrons, is much less than its effect on plasma LDL (52). This may be because VLDL and chylomicron remnants can also be effectively cleared by an LDLR-independent pathway, such as LRP1 (53). In summary, extracellular PCSK9 regulates LDLR-mediated catabolism, and intracellular PCSK9 modulates apoB secretion; the two pathways might act in a complementary fashion to regulate TG-rich lipoproteins metabolism with the extracellular pathway as the primary contributor.

### PCSK9 and Lipoprotein(a)

Elevated plasma lipoprotein(a) [Lp(a)] levels are a highly prevalent risk factor for cardiovascular disease, especially for myocardial infarction, atherosclerotic stenosis and aortic valve stenosis. Lp(a) is an apoB100–containing lipoprotein particle covalently linked to the plasminogen-like glycoprotein apo(a) by a disulfide bond (54). The statin treatment and lifestyle interventions hardly affect circulating Lp(a) levels, which brings a real challenge for successfully managing elevated Lp(a) levels in patients. Conversely, PCSK9 inhibitors dramatically reduce plasma Lp(a) levels up to ~35% in patients (54, 55). Inhibition of PCSK9 reduces the risk of coronary heart disease to a much greater degree in patients with a high plasma Lp(a) level compared to patients with a low plasma Lp(a) level (23 vs. 7%) (54). However, how PCSK9 regulates Lp(a) levels is unclear. Plasma Lp(a) levels are determined by its production and clearance. Lp(a) is removed from circulation through LDLR, SR-BI, and LRP1(54). Lp(a) levels are increased in FH patients who carry loss-of-function mutant LDLR. Overexpression of LDLR enhances Lp(a) clearance in mice. PCSK9 promotes LDLR degradation and reduces Lp(a) catabolism in HepG2 cells and primary fibroblasts (31). These findings indicate that PCSK9 can regulate plasma Lp(a) levels in a LDLR-dependent pathway. However, while statin treatment increases LDLR levels, it has no significant effect on plasma Lp(a) levels in patients. Furthermore, lymphocytes from patients with homozygous FH can effectively take up Lp(a) particles, and PCSK9 inhibitors can lower circulating Lp(a) in homozygous FH patients (56), indicating a LDLR-independent pathway. Lp(a) usually cannot compete with LDL for binding to LDLR. It has been proposed that PCSK9 inhibition can promote hepatic clearance of Lp(a) through LDLR-mediated endocytosis when plasma LDL levels are low (57). Nevertheless, although the mechanism by which PCSK9 inhibitors reduce Lp(a) levels remains to be determined, the fact that PCSK9 inhibitors provide an additional beneficial effect in lowering circulating Lp(a) may confer protection against CVD from a clinical perspective. Further work is needed to understand the role of PCSK9 in the overall metabolism of apoB-containing lipoproteins, especially for Lp(a).

### PCSK9 and Cancer Cell Immunity

MHCI on the cell surface presents specific antigens to T-cell receptors (TCR) on CD8^+^ T cells, activating CD8^+^ T cell-mediated cell killing. After antigen presentation, MHCI enters cells *via* endocytosis and is recycled to present new antigens. On the other hand, programmed cell death protein 1 receptor (PD-1) on the cell surface interacts with its ligand programmed death-ligand 1 (PD-L1) on T cells to act as an immune checkpoint, which suppresses the immune response and prevents indiscriminate attacks (58).

During tumor development, cancer cells evolve various mechanisms to escape immune attacks, such as stimulating immune checkpoint targets and reducing tumor-specific antigen (TSA) presentation. Monoclonal anti-PD1 or PDL1 antibodies, which inhibit the PD1 pathway and promote antitumor immune response, have been approved to treat various types of cancers such as melanoma, bladder cancer, non-small cell lung cancer, and renal cell carcinoma. On the other hand, MHCI on the cancer cell surface presents TSA to CD8+ cells, activating CD8+ cell-mediated cancer cell killing (58). Recently, Liu et al. reported that PCSK9 bound to MHCI on the cancer cell surface and redirected it to the lysosome for degradation, thereby reducing cell surface MHCI levels and TSA presentation. Knockout of PCSK9 or inhibition of circulating PCSK9 increased CD8^+^ T cell intratumoral infiltration and enhanced antitumor activity of CD8^+^ T cells in mice. This suppressed tumor growth of several mouse cancer cell lines, including 4T1 (breast cancer), MC38 (colon adenocarcinoma), and the PD-1 inhibitor-resistant cancer cell line, MC38R, in mice (7). In addition, knockout of PCSK9 suppressed tumor growth in *Ldlr*^−/−^ mice, indicating a LDLR-independent mechanism. However, Yuan et al. reported that inhibition of PCSK9 attenuated MC38 tumor growth in a LDLR-dependent manner. They found that LDLR directly interacted with T-cell receptor complex (TCR) and increased its cell surface levels. Inhibition of PCSK9 increased LDLR and TCR levels in MC38 tumors, enhancing TCR signaling and CD8^+^ T cell-dependent cancer cell killing in mice. The reason for the discrepancy is unclear. Yuan et al. did not report whether MHCI levels in MC38 tumors were affected by PCSK9, but they found that PCSK9 inhibition did not alter MHCI levels in B16F10 melanoma cells (59), indicating that PCSK9 regulates MHCI levels in a tumor/cell type-specific manner. These findings suggest that PCSK9 may control tumor growth through the LDLR and the MHCI pathway independently and/or collaboratively.

PCSK9 produced locally in vascular cells and cardiomyocytes can promote inflammation *via* the NF-κB signaling pathway (60, 61). Chronic inflammation increases the risk of cancer. PCSK9 expression is high in various cancers, such as hepatocellular carcinoma, gastricadenocarcinoma and prostate cancer cell lines (62–64). Zhang et al. reported that PCSK9 expression is positively correlated with poor prognosis. The authors found that PCSK9 suppressed apoptosis in cultured hepatoma-derived cell lines through the Bax/Bcl-2/Caspase9/3 pathway (64). Consistently, inhibition of PCSK9 by siRNA promotes apoptosis in a human lung adenocarcinoma cell line, A549 cells, *via* activation of caspase-3 and stimulation of ER stress (62). On the other hand, silencing PCSK9 by siRNA reduces radiation-induced apoptosis in prostate cancer cell lines, PC-3 and LnCap and thus enhances cell viability (65). However, the authors did not investigate the potential contribution of inflammation in these studies. Nevertheless, these studies suggest that PCSK9 plays a complex role in cancer development *via* different mechanisms. Cancer risk analysis of subjects carrying loss-of-function and gain-of-function mutations in PCSK9 will further reveal and confirm the role of PCSK9 in cancer progression.

## Regulation of PCSK9

PCSK9 plays a critical role in regulating circulating lipid homeostasis and MHCI-dependent immune responses. The complexity of PCSK9's functions indicates that its activity is strictly regulated by various mechanisms at multiple levels.

### Regulation of PCSK9 Expression

**Figure 2:**
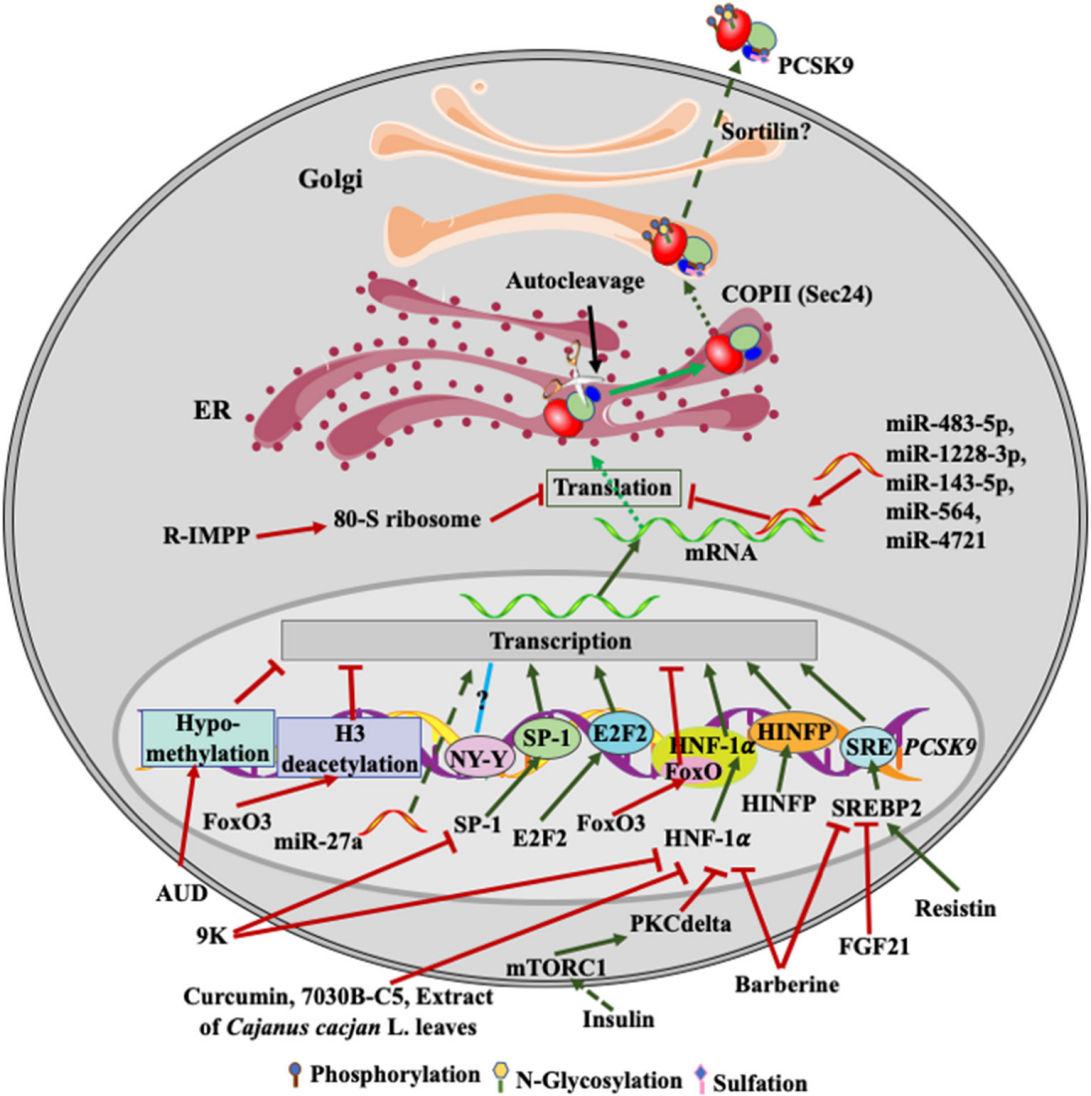
Regulation of PCSK9 expression. Transcriptional factors, such as SREBP2, HNF-1α, SP-1and E2F2, upregulate PCSK9 transcription. FGF21 and resistin inhibit and increase SREBP2-mediated transcription of PCSK9, respectively. Barberine reduces PCSK9 expression *via* suppressing the activity of SREBP2 and HNF-1α on PCSK9 transcription. 9K suppresses PCSK9 expression through SP1 and HNF-1α, while Curcumin, 7030B-C5 inhibits HNF-1α-induced transcription of PCSK9. Alcohol use causes hypomethylation of PCSK9 promoter and then reduces PCSK9 expression. Insulin activates mTROC1 and then PKCδ to suppress PCSK9 transcription *via* HNF-1α. miR-483-5p, miR-1228-3p, miR-143-5p, miR-564, and miR-4721 bind to the 3'UTR of PCSK9 mRNA, reducing PCSK9 expression, while miR27a somehow increases PCSK9 expression. R-IMPP inhibits 80S ribosome and reduces PCSK9 expression. After autocleavage in the ER, PCSK9 is transported to the Golgi *via* classical COPII vesicles. There, PCSK9 undergoes posttranslational modifications, such as phosphorylation, glycosylation, and sulfation. Mature PCSK9 is then secreted into the extra cellular environment.

Epigenetically, binding of forkhead box O (FoxO) 3 to the promoter of PCSK9 recruits Sirt6 to deacetylate histone H3, suppressing PCSK9 expression (Figure 2) (66). On the other hand, histone nuclear factor P (HINFP) binds to a HINFP motif in 20 bp upstream of the sterol regulatory element motif (SRE) in PCSK9 promoter, promoting histone H4 acetylation to activate sterol regulatory element-binding protein 2 (SREBP2)-mediated upregulation of PCSK9 transcription (67). Furthermore, *PCSK9* promoter is methylated. Alcohol use disorder (AUD) causes hypomethylation in *PCSK9* promoter and consequently reduces PCSK9 expression and plasma cholesterol levels in a mouse model of AUD, which may partially contribute to the protective effect on CVD risk observed in light alcohol users (68).

At the transcriptional level, both SREBP1 and SREBP2 have been reported to bind to SRE in PCSK9 promoter and thus upregulate PCSK9 expression in cultured cells; however, PCSK9 is predominantly regulated by SREPB2 *in vivo* (69–71). Statin treatment activates the transcriptional activity of SREBP2 and thus increases the expression of LDLR and PCSK9 (71). However, Poirier et al. reported that the expression of PCSK9 in the rodent central nervous system was regulated in a SREBP2-independnet manner (72). *PCSK9* promoter also contains a binding site of the transcription factor hepatocyte nuclear factor 1 alpha (HNF1α) (70, 73). Silencing of HNF1α but not HNF1β significantly reduced PCSK9 expression. Furthermore, insulin increases the mTORC1 signaling pathway and activates PKCδ, which reduces HNF1α-mediated expression of PCSK9 and increases hepatic LDLR levels (74). Li et al. reported that HNF1α worked cooperatively with SREBP2 to activate PCSK9 transcription since mutations in the HNF1α-binding site significantly reduced SREBP2-mediated upregulation of PCSK9 transcription (73). However, the HNF1α binding site is just 28 bp upstream of the SREBP2 bindings site. Mutations in the HNF1α binding site may affect the integrity of SRE and then indirectly impair SREBP2 binding.

The HNF1α binding site in the promoter of *PCSK9* contains a consensus FoxO binding site (Figure 2). FoxO3 can inhibit PCSK9 expression competitively *via* inhibiting HNF1α-mediated upregulation of PCSK9 (66). In addition, Lai et al. reported that transcription factor E2F2 could bind to the *PCSK9* promoter region and upregulate its expression under a condition of feeding or high cellular cholesterol levels (75). The promoter region of PCSK9 also contains NF-Y and SP1 binding sites upstream of SRE (71). The putative NF-Y binding site appears not to affect PCSK9 expression. However, the SP1 site may mediate basal transcription of PCSK9 since it is not required for the sterol-dependent regulation of PCSK9 expression, but mutations in this site reduce PCSK9 expression (69). A variant, C-332C>A, in the SP1 binding site, increases PCSK9 expression by approximately 2.5-fold independent of lovastatin treatment (76).

Several lines of evidence demonstrate the regulation of *PCSK9* transcription by small molecules (Figure 2). Curcumin and the methanol extract of *Cajanus cajan* L. leaves reduce HNF1α levels and downregulate PCSK9 transcription in HepG2 cells (77, 78). Epigallocatechingallate (humans, Sprague-Dawl rats, HepG2 and Huh7 cells), ascorbic acid (mice, HepG2 and Huh7 cells), Pinostrobin (HepG2 cells), and tanshinone IIA (HepG2 cells) reduce PCSK9 expression in a FoxO3a-dependent manner, probably *via* attenuating HNF1α-mediated activation of PCSK9 expression and/or methylation in the promoter region of PCSK9 (79–82). A small molecular, 7030B-C5, also reduces PCSK9 expression in HepG2 cells and mice mainly through the FoxO1 and HNF1α pathway (83). In addition, Berberine reduces PCSK9 expression mainly through attenuating SREBP2 and HNF1α-mediated upregulation of PCSK9 transcription in HepG2 cells (73), which may account for its cholesterol-lowering effect. Conversely, a berberine derivative, 9k, downregulates PCSK9 expression *via* suppressing the transcriptional activity of HNF1α and/or SP1 in HepG2 cells (84). Fibroblast growth factor 21 inhibits the transcriptional activity of SREBP2, thereby reducing PCSK9 expression in mouse liver (85). In addition, glucagon, bile acids, fibrate, and oncostatin M have been reported to inhibit PCSK9 expression at the transcriptional levels in HepG2 cells (86–88), but the underlying mechanisms are unclear. On the other hand, resistin, a small cysteine-rich protein secreted from macrophages and adipose tissues, increases PCSK9 transcription *via* the SREBP2 pathway in HepG2 cells and primary human hepatocytes (89). Nevertheless, these findings indicate the potential of inhibiting PCSK9 transcription as an avenue to lower plasma cholesterol levels, reducing CVD risk. However, the aforementioned transcriptional factors also regulate the transcription of many other proteins that play important roles in various physiological processes. For example, inhibition of SREBP2 reduces LDLR expression, attenuating LDL clearance. Inhibition of HNF1α activity does not affect LDLR expression. However, HNF1α can act as a tumor suppressor, and its expression is reduced in patients with liver malignancies (90). Thus, it is a big challenge to develop small molecules that can specifically modify PCSK9 expression at the transcriptional level.

Post-transcriptionally, the expression of PCSK9 is regulated by microRNAs (miRNA) (Figure 2). miR-483-5p targets the 3′-UTR of the PCSK9 mRNA, reduces PCSk9 expression and decreases plasma cholesterol levels in HepG2 cells and mice (91). Similarly, miR-224, miR-191, miR-222, miR-1228-3p, miR-564, miR-4721, miR-337-3p, and miR-143-5p can reduce PCSK9 expression through targeting its 3′-UTR in cultured cells, such as Huh7, HepG2 and BON-1 cells (92–94). A common variant, 1420C>G, decreases the inhibitory effect of miR-1228-3p and miR-143-5p on PCSK9 expression, reducing plasma levels of PCSK9 and LDL cholesterol (95). Similarly, Los et al. identified several variants in *PCSK9* 3′-UTR in FH patients. The variant 345C>T impairs binding of miR-4721 and miR-564 to *PCSK9* 3′-UTR and increases PCSK9 expression (93). Conversely, miR-27a upregulates PCSK9 expression, possibly through binding to the upstream of PCSK9 promoter in HepG2 cells (96). It is of note that a single miRNA often targets multiple genes as binding of miRNAs to their target genes requires seed pairing of as few as six nucleotides or even imperfect seed pairing (97). Thus, one of the key issues of miRNA-based therapies is their potential off-target effect.

Compared to miRNA, siRNAs bind to their complementary sequence in mRNA that completely matches their antisense strand, thereby specifically reducing the expression of their target genes. Phase III trials of Inclisiran, a chemically modified siRNA that targets PCSK9 mRNA, shows a promising lipid-lowering effect. Subcutaneous injection of Inclisiran reduces plasma LDL-C levels up to 50% in heterozygous FH patients without any major side-effects (26). Inclisiran, which requires twice-yearly administration, may reduce the cost of PCSK9 inhibitors compared to the current PCSK9 monoclonal antibody therapy that needs administration every 2–4 weeks. However, it is still a financial burden as a primary prevention measure for all eligible patients. In addition, siRNAs, particularly at a high dose, also exhibit miRNA-like off-target activity (98). Additionally, duplex siRNA can trigger an innate immune response in Toll-like receptors-dependent and independent mechanisms (99). Patients with Inclisiran treatment do show a slightly increased rate of mild-to-moderate bronchitis (4.3 vs. 0.7% for Inclisiran and placebo, respectively) (26). Therefore, possible long-term side effects of using siRNAs as a lifelong primary prevention strategy need to be assessed.

A small molecule, R-IMPP, can selectively target human 80S ribosome and inhibit PCSK9 translation. R-IMPP significantly reduces the protein level of PCSK9, thereby increasing LDLR levels and LDL uptake in Huh7 cells (100). However, the therapeutic potential of R-IMPP is uncertain because ribosomes are the core of protein translation machinery and not an ideal therapeutic target.

Most recently, Liu et al. reported that the blood flow rate regulated PCSK9 expression through the toll-like receptor 4-MyD88-NF-κB signaling pathway in the rabbit thoracic aorta. Low-flow state increased, whereas high-flow state reduced the mRNA and protein level of PCSK9 in vascular cells. Interestingly, they observed an opposite effect on the expression of LDLR (101), indicating that the impact of flow rate is independent of SREBP2. Interestingly, knockdown of PCSK9 suppressed, while overexpression of PCSK9 enhanced the toll-like receptor 4-NF-κB signaling pathway and inflammation in the atherosclerotic lesions of apoE^−/−^ mice (60). It will be of interest to see if the increased expression of PCSK9 under the low-flow state promotes the toll-like receptor 4-NF-κB signaling pathway in the thoracic aorta.

Circulating PCSK9 is mainly produced by hepatocytes. Blood flow rate may not have a similar effect on PCSK9 expression in hepatocytes since hepatocytes, unlike aortic vascular cells, are not directly exposed to blood flow. On the other hand, blood flow rate equals blood flow divided by cross-section area. Atherosclerosis causes blood vessels to harden and to narrow, which increases blood flow rate. It would be of interest to assess whether PCSK9 expression in vascular cells near and at atherosclerotic lesion area is reduced due to the increased blood flow rate, which could lead to a beneficial outcome since vascular cell PCSK9 can promote inflammation.

### Regulation of PCSK9 Secretion

Although multiple tissues express PCSK9, circulating PCSK9 is mainly secreted from the liver (102). Loss-of-function PCSK9 mutations such as G236S, S462P, and C679X reduce its secretion, while gain-of-function mutations such as E32K enhance PCSK9 secretion (17, 103). Furthermore, circulating PCSK9 is rapidly cleared from the blood with a half-life of about 5 min in mice (37), indicating that targeting PCSK9 secretion is a promising therapeutic strategy. However, the machinery system controlling PCSK9 secretion is still elusive.

PCSK9 undergoes autocleavage in the ER (104), which is essential for its maturation and secretion. However, the enzymatic activity is not required for this processing. Mutant PCSK9 that losses its catalytic activity is retained in the ER, but coexpression of prodomain with catalytic dead mutant PCSK9 rescues its secretion in HepG2 cells (29). In addition, deletion of part of the C-terminal domain of PCSK9 impairs its secretion but does not affect its autocleavage in cultured human hepatocytes, such as HepG2 and Huh7 cells (17, 105), indicating that the autocleavage is not sufficient to support PCSK9 secretion. After autocleavage, the cleaved N-terminal PRO is associated with the catalytic domain and functions as an intramolecular chaperone, guaranteeing the correct folding of the catalytic domain in the ER. This step is believed to be the rate-limiting step for PCSK9 mature and secretion (103).

PCSK9 is transported from the ER to the Golgi *via* the classical COPII vesicles. The lack of SEC24, one of COPII components, significantly reduces PCSK9 secretion in mice and cultured human hepatocytes, HepG2 and Huh7 cells (17, 106). However, PCSK9 is a secretory protein located in the ER lumen, while SEC24 is located in the cytosol. Therefore, a cargo receptor is required to bridge the interaction between PCSK9 and SEC24. Emmer et al. reported that a cargo receptor Surf4 facilitated secretion of PCSK9 that was overexpressed in HEK293 cells. They found that Surf4 co-immunoprecipitated with PCSK9, and knockout of Surf4 significantly reduced the amount of PCSK9 detected in culture medium (107). Surf4 is a transmembrane protein that mainly resides in the ER membrane. It contains five putative transmembrane domains, an ER lumen-exposed N-terminus that binds cargo proteins within the lumen, and a cytoplasmic domain that interacts with COPII components, facilitating cargo sorting into COPII vesicles. However, we found that knockdown of Surf4 in cultured immortalized human hepatocytes, HepG2 and Huh7 cells, did not impair endogenous PCSK9 secretion (108). This discrepancy may be caused by different types of cells used in the two studies. We investigated endogenous PCSK9 secretion from cultured hepatocytes, HepG2 and Huh7 cells, while Emmer et al. studied the effect of Surf4 on secretion of PCSK9 overexpressed in HEK293 cells that do not express endogenous PCSK9. Furthermore, we found that knockdown of Surf4 in mouse liver had no significant effect on plasma and hepatic PCSK9 levels. In liver-specific Surf4 knockout mice, the levels of PCSK9 in plasma and liver homogenate were also comparable to that in the wild-type mice (109). Therefore, Surf4 is not required for endogenous PCSK9 secretion.

The C-terminal domain of PCSK9 has been implicated in its secretion. Loss-of-function mutations such as E498K and S462P located in the C-terminus of PCSK9 damage its secretion (12, 103). Deletion of the entire C-terminal PCSK9 from amino acids 456 to 692 does not impair PCSK9 secretion. Conversely, removing part of the C-terminal region (from amino acids 457 to 528 or 608 to 692) damages PCSK9 secretion (16, 17, 105). Furthermore, the deletion of part of or the entire hinge region that connects the catalytic domain and the C-terminal domain also significantly reduces PCSK9 secretion, indicating the important role of this region. SEC24 silencing significantly reduces the secretion of the wild-type but not mutant PCSK9 without the C-terminal domain, indicating that the C-terminal region of PCSK9 may be involved in SEC24-facilitated PCSK9 secretion (17). Further studies are required to elucidate how PCSK9 is sorted into COPII vesicles. Most recently, Rogers et al. reported that dynamin-related protein1 (DRP1)-mediated ER remodeling involved in PCSK9 secretion (110). Inhibition of DRP1 by mitochondrial division inhibitor 1 or knockout hepatic DRP1 markedly reduced PCSK9 secretion in HepG2 cells and mice.

After delivery to the Golgi apparatus, PCSK9 undergoes various posttranslational modifications and is then packed into secretory vesicles. The vesicles are delivered to and fused with the plasma membrane, releasing PCSK9 into the extracellular milieu. Gustafsen et al. reported that sortilin co-immunoprecipitated with PCSK9, and the two proteins were colocalized in the trans-Golgi network in HepG2 cells. Knockout of sortilin significantly reduced plasma PCSK9 levels in mice and reduced PCSK9 secretion from mouse primary hepatocytes. The author further showed that PCSK9 levels were positively correlated with sortilin levels in human serum. Thus, they concluded that sortilin interacted with PCSK9 in the trans-Golgi network, facilitating PCSK9 secretion (111). On the other hand, Butkinaree et al. reported that plasma levels of PCSK9 were comparable in sortilin knockout mice and wild-type littermates, and sortilin had no effect on PCSK9-promoted LDLR degradation in HepG2 and Huh7 cells. Instead, PCSK9 induced sortilin degradation (35). The reason for this discrepancy is unclear. The mice used in the Gustafsen study were C57BL/6J background, while Butkinaree et al. did not report their mouse background. Nevertheless, these findings reveal the complexity of the molecular mechanisms of PCSK9 secretion.

### Posttranslational Modifications of PCSK9

PCSK9 is predicted to be phosphorylated on serine, threonine, asparagine, and lysine residues by PhosphoSitePlus (www.phosphosite.org). Mass spectrometry analysis of plasma samples confirms this prediction. Furthermore, Dewpura et al. reported that PCSK9 was partially phosphorylated on serine residues at positions 47 and 688 by a Golgi casein kinase-like kinase in a cell-type dependent manner, with the highest phosphorylation in HepG2 cell (~70%), followed by Huh 7 cells (~54%), HEK293 cells (~23%), and CHO-K1cells (none). Phosphorylation may protect PCSK9 against proteolysis and increase its stability in Huh 7 cells (112). Meanwhile, this finding also indicates that serine phosphorylation is not required for PCSK9's action on LDLR since PCSK9 purified from CHO-K1 cells is unphosphorylated and can effectively promote LDLR degradation (38). It has also been reported that PCSK9 was phosphorylated on serine residues at positions 47, 666, 668, and 688 by family with sequence similarity 20, member C (FAM20C), which increased PCSK9 secretion and enhanced its ability to promote LDLR degradation in HepG2 cells (113). However, phosphorylation at these sites was also not required for PCSK9-promoted LDLR degradation since mutant PCSK9 that lost phosphorylation at the four serine residues still could stimulate LDLR degradation. In addition, FAM20C phosphorylates serine residue in a consensus Ser-x-Glu motif present in many secreted proteins (114). Thus, it cannot be ruled out that FAM20C may indirectly affect PCSK9 secretion and function *via* phosphorylation of other proteins.

PCSK9 is N-glycosylated on asparagine residue at position 533 and sulfated on tyrosine residues (104). Detailed analysis revealed that the N-glycosylation at Asn533 and sulfation at Tyr38 were not required for PCSK9 processing, secretion and function in HepG2 and Huh 7 cells (115). Treatment of cells with tunicamycin that inhibits N-glycosylation or chlorate that inhibits tyrosine sulfation had no effect on PCSK9 expression and secretion in the human hepatocyte cell line, Huh7 cells. When overexpressed, mutant PCSK9 that lost the N-glycosylation and Tyr sulfation sites alone or together could be efficiently secreted and promote LDLR degradation in Huh7 and HepG2 cells (116). However, the secreted mutant PCSK9 appeared to promote LDLR degradation less effectively than the wild-type protein, suggesting that N-glycosylation may enhance the ability of PCSK9 to stimulate LDLR degradation.

Plasma levels of PCSK9 in subjects carrying phosphomannose mutase 2 (PMM2) variants (p.R141H and p.P69S) are significantly reduced by approximately 42% compared to the controls, which might contribute to hypolipidemia observed in these patients (116). The PMM2 variants may affect N-glycosylation of PCSK9 and its secretion *in vivo*, even though removal or inhibition of N-glycosylation in PCSK9 does not affect its secretion in cultured cells. Alternatively, these variants may impair PCSK9 secretion indirectly by affecting unknown factors that are important for PCSK9 secretion since PMM2 is required for the synthesis of GDP-mannose, a mannose donor for N-glycosylation. Analysis of secretion of N-glycosylation defective PCSK9 mutant in *Pcsk9*^−/−^ mice might provide a clue for the potential role of N-glycosylation in PCSK9 secretion *in vivo*. Nevertheless, these studies indicate that posttranslational modifications, including phosphorylation, sulfation and N-glycosylation, may affect but are not required for PCSK9 processing, secretion, stability and function.

## Conclusion and Perspectives

PCSK9 regulates plasma cholesterol levels and tumor-specific antigen presentation primarily through promoting LDLR and MHCI degradation, respectively. Of note, the lack of PCSK9 in humans does not cause any known notable side effects (3, 6). Therefore, PCSK9 is a promising therapeutic target to reduce the risk of the top two leading causes of mortality worldwide, cardiovascular disease and cancer. Various strategies have been or are being developed to inhibit PCSK9 (117–120) (Table 1). Current PCSK9 inhibitors, evolocumab and alirocumab, are humanized monoclonal anti-PCSK9 antibodies. They can significantly reduce plasma LDL cholesterol levels and cardiovascular events in patients with hypercholesterolemia and suppress tumor growth in mice. Inclisiran, a chemically synthesized siRNA targeting PCSK9 mRNA, also significantly reduces plasma LDL cholesterol levels by about 30–50% with only two injections each year. However, these strategies are expensive, limiting their widespread use. Other strategies, such as CRISPR-Cas9 gene editing and PCSK9 vaccine, are only in preclinical studies or phase I clinical trials (119, 120). Therefore, there is an urgent need for further research to elucidate the underlying mechanisms for PCSK9's impact on lipid metabolism and cancer growth. For example, (1) PCSK9 binds to LDLR with a much higher affinity at the acidic endosomal environment to block LDLR recycling. Does PCSK9 bind to MHCI in a pH-dependent manner? (2) PCSK9, LDLR and MHCI do not contain a lysosomal targeting signal; how is PCSK9/LDLR and PCSK9/MHCI complex redirected from the endosome to the lysosome for degradation? (3) Circulating PCSK9 is mainly secreted from hepatocytes and then promotes LDLR and MHCI degradation. What machinery system assists PCSK9 secretion? (4) HSPG facilitates PCSK9-promoted hepatic LDLR degradation. Is there a cofactor assisting PCSK9's action on MHCI? Answering these questions is critical to the development of innovative and more cost-effective treatment options to inhibit PCSK9-promoted degradation of LDLR and MHCI.

**Table 1:** PSCK9 inhibitors.

**Name**	**Strategy**	**Target**	**Mechanism**	**Status**
Evolocumab	Humanized monoclonal antibody	CAT of PCSK9	Blocking PCSK9 binding to LDLR	Approved
Alirocumab	Humanized monoclonal antibody	CAT of PCSK9	Blocking PCSK9 binding to LDLR	Approved
Inclisiran	GalNAc-conjugated siRNA	mRNA of PCSK9	Inhibiting PCSK9 expression	Under review by the FDA
LIB003	Adnectin-human serum albumin fusion protein	CAT of PCSK9	Blocking PCSK9 binding to LDLR	Phase III
AT04A and AT06A	PCSK9 peptide Vaccine	Aa 153-162 of PCSK9	Blocking PCSK9 binding to LDLR	Phase I
Mimetic peptide	Mimicking the binding site of PCSCK9 on LDLR	CAT of PCSK9	Blocking PCSK9 binding to LDLR	Preclinical
DRP	Small PCSK9 inhibitor		Inhibiting interaction between PCSK9 and HSPG	Preclinical
NYX-330	Small PCSK9 inhibitor	PCSK9	Inhibition of PCSK9 binding to LDLR	Preclinical
PF-0644846	Inhibitor of ribosome	80S ribosome	Inhibition of *PCSK9* translation	Preclinical
CRISPR-Cas9	Gene editing	PCSK9 gene	Knockout/knockdown of PCSK9 expression	Preclinical
9k	Small inhibitor, berberine derivative	The HNF1α pathway	Inhibition of *PCSK9* transcription	Preclinical
7030B-C5	Small inhibitor	the FoxO1 and HNF1α pathway	Inhibition of *PCSK9* transcription	Preclinical

## Author Contributions

X-dX and Z-sP wrote the initial draft. G-qW and D-wZ supervised the final version. H-mG participated in the discussion and the preparation of the manuscript. MW reviewed and edited the manuscript. All authors contributed to the article and approved the submitted version.

## Funding

This study was supported by the Natural Sciences and Engineering Research Council of Canada (RGPIN-2016-06479) and Canadian Institutes of Health Research (PS 155994). X-dX and G-qW were supported by funding from Qingyuan People's Hospital.

## Conflict of Interest

The authors declare that the research was conducted in the absence of any commercial or financial relationships that could be construed as a potential conflict of interest.

## Publisher's Note

All claims expressed in this article are solely those of the authors and do not necessarily represent those of their affiliated organizations, or those of the publisher, the editors and the reviewers. Any product that may be evaluated in this article, or claim that may be made by its manufacturer, is not guaranteed or endorsed by the publisher.
